# Activation of Wnt/Beta-Catenin Signaling Pathway as a Promising Therapeutic Candidate for Cerebral Ischemia/Reperfusion Injury

**DOI:** 10.3389/fphar.2022.914537

**Published:** 2022-05-20

**Authors:** Zhizhun Mo, Zhongyi Zeng, Yuxiang Liu, Linsheng Zeng, Jiansong Fang, Yinzhong Ma

**Affiliations:** ^1^ Emergency Department, Shenzhen Traditional Chinese Medicine Hospital, Shenzhen, China; ^2^ Science and Technology Innovation Center, Guangzhou University of Chinese Medicine, Guangzhou, China; ^3^ Shenzhen Key Laboratory of Biomimetic Materials and Cellular Immunomodulation, Institute of Biomedicine and Biotechnology, Shenzhen Institute of Advanced Technology, Chinese Academy of Sciences, Shenzhen, China

**Keywords:** ischemic stroke, ischemia/reperfusion injury, Wnt signaling, neuroprotection, blood-brain barrier

## Abstract

Stroke is one of the leading causes of mortality, and survivors experience serious neurological and motor behavioral deficiencies. Following a cerebral ischemic event, substantial alterations in both cellular and molecular activities occur because of ischemia/reperfusion injury. Wnt signaling is an evolutionarily conserved signaling pathway that has been manifested to play a key role in embryo development and function maintenance in adults. Overactivation of Wnt signaling has previously been investigated in cancer-based research studies. Recently, abnormal Wnt signaling activity has been observed in ischemic stroke, which is accompanied by massive blood–brain barrier (BBB) disruption, neuronal apoptosis, and neuroinflammation within the central nervous system (CNS). Significant therapeutic effects were observed after reactivating the adynamic signaling activity of canonical Wnt signaling in different cell types. To better understand the therapeutic potential of Wnt as a novel target for stroke, we reviewed the role of Wnt signaling in the pathogenesis of stroke in different cell types, including endothelial cells, neurons, oligodendrocytes, and microglia. A comprehensive understanding of Wnt signaling among different cells may help to evaluate its potential value for the development of novel therapeutic strategies based on Wnt activation that can ameliorate complications and improve functional rehabilitation after ischemic stroke.

## Introduction

Stroke is the leading cause of disability and mortality worldwide and is classified as ischemic or hemorrhagic. Ischemic stroke accounts for 87% of all stroke incidences and is defined as the interruption of blood flow to the brain due to blockage of the cerebral artery, causing severe damage to focal brain tissue (Collaborators, 2019). Patients who survive the initial ischemic attack often suffer from associated complications, such as hemiparesis, cognitive deficits, and dependency in daily activities, the rehabilitation of which has always been a challenging issue (Richards et al., 2015). According to an estimation put forth by the American Heart Association/American Stroke Association, the total economic cost to the society for stroke is likely to rise up to $184.1 billion for the year of 2030 (Collaborators, 2019).

Vascular recanalization remains the primary therapeutic option (Powers et al., 2019). To date, thrombolytic therapy with intravenous recombinant tissue-type plasminogen activator (rtPA) is recommended within the first 3–4.5 h. Thrombolysis beyond the time window has a certain recanalization ability, but the main side effects of intracerebral hemorrhage increase concomitantly (Emberson et al., 2014). As for embolization of larger vessels (anterior circulatory arterial occlusion), which accounts for one-fourth of all ischemic strokes, intravenous thrombolysis has a low recanalization rate of about 13–20% (Ma et al., 2019). Therefore, rtPA thrombolysis paired with mechanical thrombectomy has become the first line therapy for large vessel occlusion. According to the American Heart Association, mechanical thrombectomy is prescribed in cases where indications of middle cerebral artery embolism or internal carotid artery embolism are evident within 6 h of symptom onset (Alawieh et al., 2017). More recently, screening by imaging methods has shown that patients with penumbra can also benefit from mechanical thrombectomy 16–24 h after the onset of symptoms (Albers et al., 2018). Additionally, to achieve recanalization as soon as possible, there are studies on mechanical thrombectomy that skip intravenous thrombolysis (DIRECT-MT, DEVT) and show non-inferiority (Yang et al., 2020). In addition to vascular recanalization therapy, the treatment of acute ischemic stroke includes antiplatelet and anticoagulant therapies, improvement of microcirculation, lipid control, and neuroprotection (Phipps and Cronin, 2020).

The above studies suggest that the ischemic penumbra still has great therapeutic potential for neuroprotection. The Wnt pathway is part of an evolutionarily conserved intracellular signal transduction cascade that regulates multiple processes crucial for cell proliferation, differentiation, migration, and fate decision during development (Schulte and Bryja, 2017; Eubelen et al., 2018). Recently, several studies have reported the mechanism by which Wnt/β-catenin signaling is regulated in the adult brain and serves as an endogenous protective mechanism against the central nervous system (CNS) diseases (Schulte and Bryja, 2017; Menet et al., 2020; Cheng et al., 2022). In this review, we discuss the recent research updates on the regulatory mechanism of the classical Wnt (Wnt/β-catenin) signaling pathway and summarize the biological functions of the cells (endothelial cells, neurons, oligodendrocytes, microglia, and astrocytes) affected by stroke pathology. Furthermore, various therapeutic studies targeting the Wnt/β-catenin signaling pathway have been conducted. This review provides insights into the potential and the value of the Wnt/β-catenin signaling pathway as a therapeutic target for ischemic stroke.

## Acute Pathology in Post-Ischemic Stroke

**FIGURE 1:**
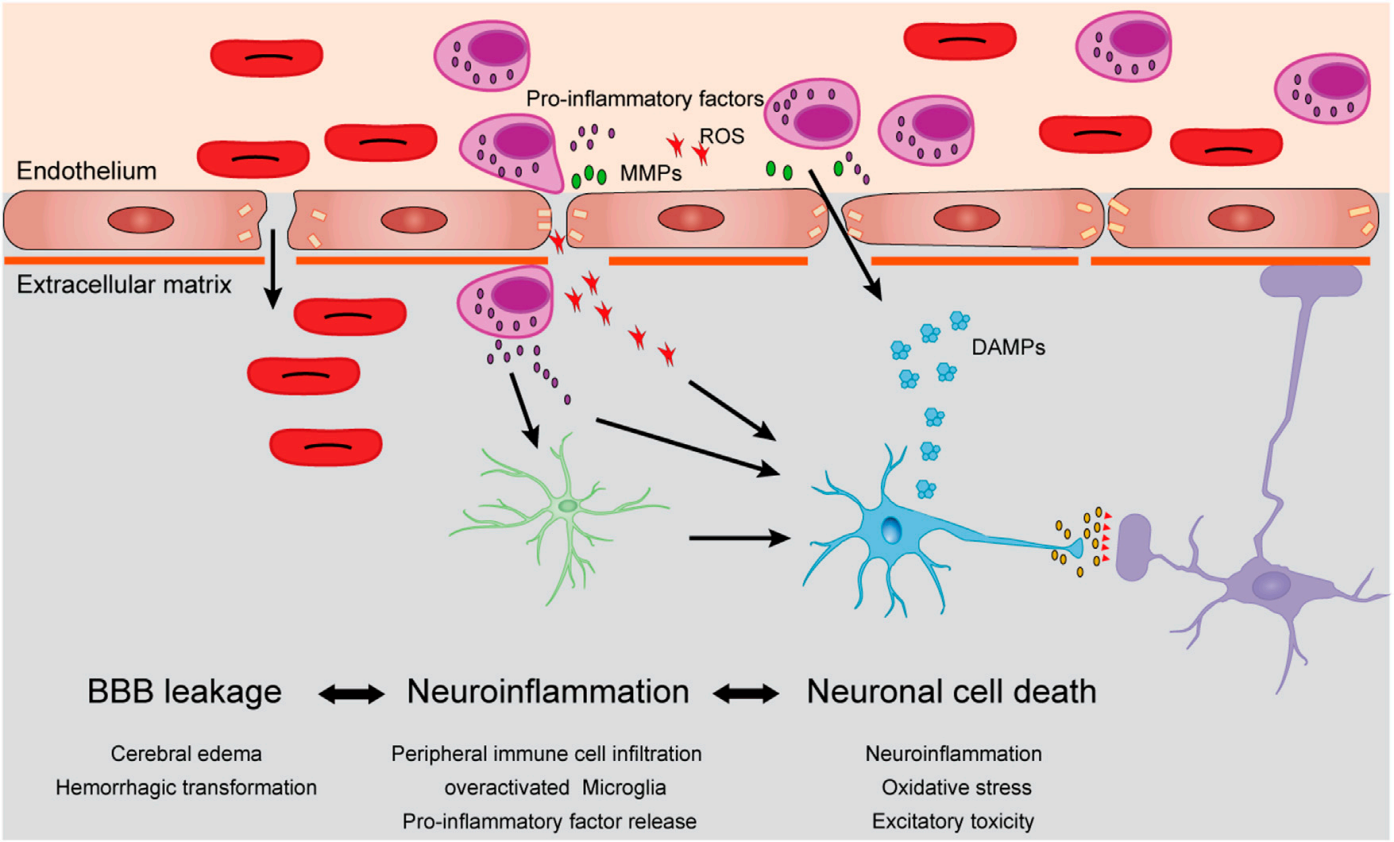
Cellular mechanisms of cerebral ischemia/reperfusion injury. After stroke, dying cells from ischemic brain tissue begin to produce damage-related molecular patterns (DAMPs), which induced circulating neutrophils to infiltrate into ischemic brain parenchyma. Neutrophils released matrix metalloproteinases (MMPs), reactive oxygen species (ROS) and pro-inflammatory factors, accelerate their infiltration and damage the vascular endothelial cells and extracellular basal membrane, which induced brain edema and even hemorrhagic transformation. A large number of inflammatory factors and ROS can also stimulate the overactivation of microglia, which further aggravates neuronal damage caused by neuroinflammation and leads to neuronal death.

Several studies suggest that a series of biochemical reactions occur within a few minutes after ischemia/reperfusion, causing strong oxidative stress and excitotoxic damage to the brain tissue (Chamorro et al., 2016). Meanwhile, circulating immune cells (mostly neutrophils) rapidly adhere to the endovascular cortex of the ischemic region and infiltrate the brain parenchyma by releasing proteolytic enzymes and matrix metalloproteinases (MMPs) to affect the integrity of the blood–brain barrier (BBB) (Wang et al., 2021a). The innate immune mechanism of neutrophils can also release large amounts of reactive oxygen species (ROS) through respiratory burst which may damage vascular endothelial cells (Wang et al., 2021a). Days after the primary stroke and transient ischemic attack, more circulating immune cells (monocytes/macrophages and T lymphocytes) enter the brain parenchyma, and along with local microglia, release a large number of inflammatory factors, such as tumor necrosis factor α (TNF-α), interleukin 1-β (IL-1β), and interleukin 6 (IL-6), which cause serious inflammatory damage to the glia and neurons, ultimately leading to neuronal apoptosis and necrosis (Wang et al., 2021b; Qiu et al., 2021). Large amounts of dead cell debris form damage-associated molecular patterns (DAMPs), which further activate the immune response and cause damage to the brain tissue (Figure 1).

## Progress in Drug Development Toward Ischemic Stroke

Owing to the complexity of the physiological and pathological mechanisms of the human brain, drug development to ameliorate ischemic stroke is acknowledged as a very challenging task. Thousands of lead compounds that show promising therapeutic effects in preclinical trials rarely show sufficient efficacy during the trial phases (O'Collins et al., 2006). In 2020, a combination of Edaravone and Dexcamphenol (Xianbixin) showed promising results for the treatment of acute ischemic stroke in clinical trials. Innovation of the therapeutic strategy relies on powerful dual targets against free radicals and inflammation. The only new drug approved for stroke over the past 5 years, Xianbixin, which blocks cascading damage in the brain tissue during ischemia/reperfusion injury, provides new insights into the development of drugs for ischemic stroke. Therefore, signaling pathways that exist in a variety of brain cells and exert their corresponding protective effects will be more suitable as drug targets for treatment of stroke.

## The Intracellular Trafficking of Wnt Signaling

Previous studies in drug development have shown that a single protective mechanism cannot completely block the cascading damage after ischemic stroke; therefore, it is difficult to achieve adequate therapeutic effects (Moskowitz et al., 2010; Zhou et al., 2018). The treatment for stroke requires multiple protective mechanisms. In recent years, the Wnt signaling pathway has been shown to play an important regulatory role in maintaining cerebrovascular and neural cell functions (Menet et al., 2020). The Wnt signaling pathway is widely found in invertebrates and vertebrates and is a highly evolutionarily conserved signaling pathway. It plays a crucial role in the early development of embryos, organogenesis, and maintenance of normal physiological functions in adults (Schulte and Bryja, 2017; Jean LeBlanc et al., 2019; Routledge and Scholpp, 2019).

Wnt signaling is a complex regulatory network consisting of two branches: canonical and non-canonical pathways (Eubelen et al., 2018; Routledge and Scholpp, 2019). The canonical Wnt signaling pathway, also known as the Wnt/β-catenin signaling pathway, begins with the binding of the ligand of Wnt protein with the receptors of Frizzled (FZD) and low-density lipoprotein receptor-related proteins 5 and 6 (LRP5/6), which activates a series of complex biochemical reactions and blocks the cytoplasmic β-catenin degradation pathway, thereby enabling β-catenin accumulation in the cytoplasm. After accumulation in the nucleus, β-catenin assembles with T cytokines (TCF/LEF) to form a transcription complex, which ultimately regulates the expression of target genes (Routledge and Scholpp, 2019).

**FIGURE 2:**
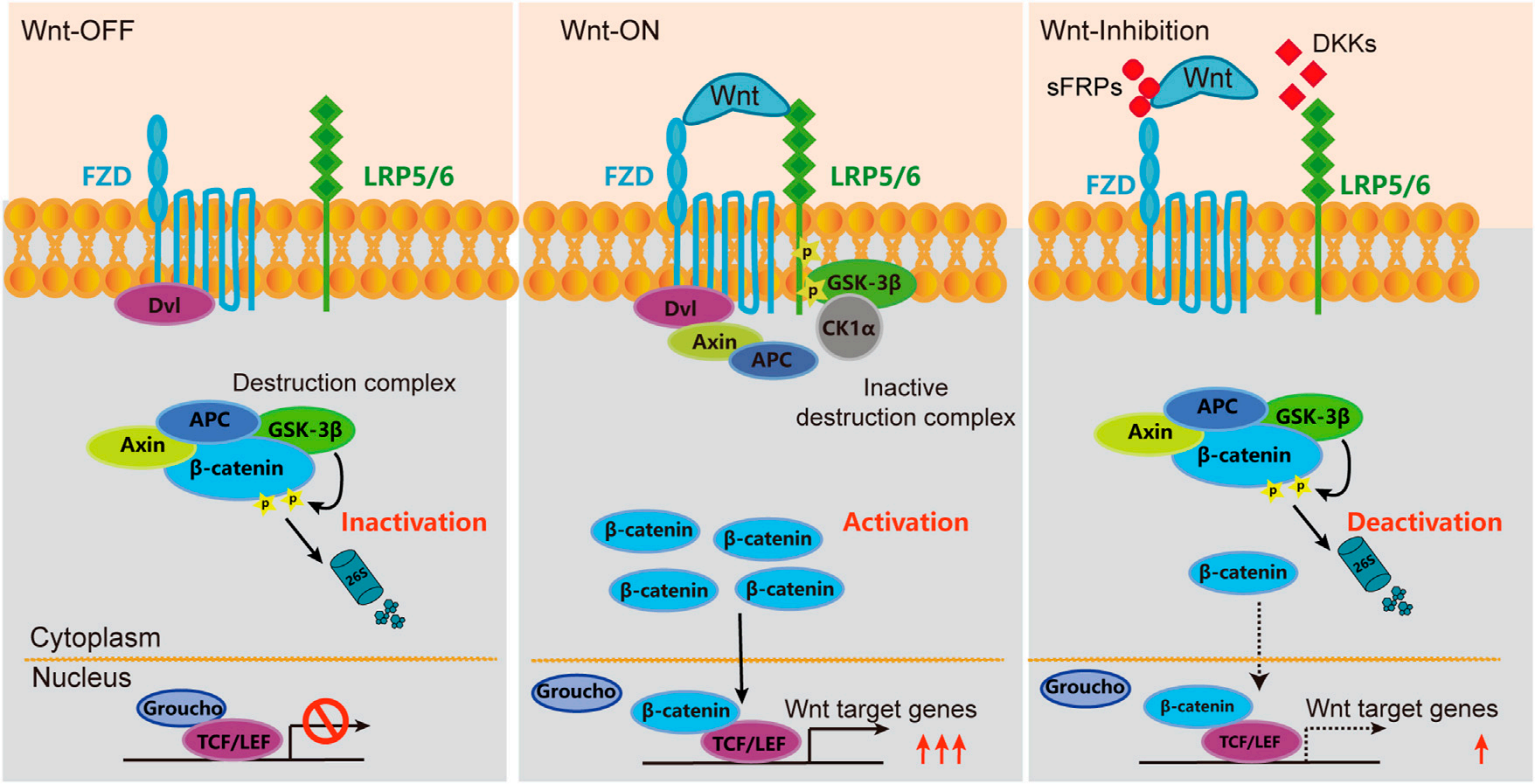
Schematic diagram of Wnt/β-catenin signaling pathway in inactivated, activated and deactivated states. In the quiescent Wnt/β-catenin-dependent pathway (Wnt Off), β-catenin undergoes continuous ubiquitination in the absence of Wnt protein by the destruction complex. In this state, Wnt target genes are suppressed by Groucho and TCF/LEF transcription factors. Upon Wnt binding to FZD receptors and the co-receptor Lrp5/6 and formed a ligand-receptor complex called the “signalosome,” which further recruit the intracellular Dvl and components of the destruction complex to the cell membrane (Wnt On). This would prohibit the formation of destruction complex and thus prevents the degradation of β-catenin and allowing its nuclear translocation. β-catenin would subsequently bind with TCF/LEF transcription factors to inhibit their DNA binding. Wnt target genes, such as *Axin2* and *Nkd1*, are disinhibited to transcript. Some extracellular molecules could inhibit the formation of signalosome. For example, DKKs can competitively bind to LRP6, while sFRPs can directly bind to Wnt proteins and reduce the activity of signal transduction.

Wnt proteins were discovered 30 years ago, with 19 Wnt proteins clustered into 12 subfamilies and distributed in tissues and organs across the body (Clevers and Nusse, 2012). It is noteworthy that in addition to Wnt proteins, the activity of the Wnt/β-catenin signaling pathway is also regulated by a variety of extracellular signaling molecules, such as Dickkopf proteins (DKK1-4; competitively inhibits Wnt proteins by binding to LRP5/6) and secreted Frizzled-related proteins (sFRPs; inhibits Wnt signaling by directly binding to Wnt protein) (Wang et al., 2000; Zorn, 2001). Seib et al. found that the expression of *Dkk1* gene in mouse neural stem cells in the sub granular zone increased with age and inhibited Wnt signaling activity, while neural stem cell-specific *Dkk1* knockout significantly increased Wnt signaling activity and adult neurogenesis in aged mice (Seib et al., 2013). Similarly, Zhu et al. found that the expression of *Dkk3* in neural stem cells in the subventricular zone was upregulated with age, and neurogenesis and olfactory function were downregulated in aged mice (Zhu et al., 2014). In another study, it was found that sFRP3 is highly expressed in the dentate gyrus and inhibits the proliferation of neural stem cells in the sub granular zone (Jang et al., 2013). Cho et al. showed that the knockdown of sFRP3 in the dentate gyrus significantly improved adult neurogenesis in a mouse model of premature aging induced by the mitotic checkpoint kinase *BubR1* gene mutation (Cho et al., 2019). The extracellular and intracellular Wnt/β-catenin signaling pathways are shown in Figure 2.

## Implications of Wnt/β-Catenin Signaling Pathway Within Neural Vascular Unit During Ischemic Stroke

The CNS, including the brain and spinal cord, is characterized by a highly active metabolism and high sensitivity to extraneous substances. To maintain normal function and the microenvironment, blood vessels within the CNS have special functions that other tissue vessels do not. A notable function is maintaining the blood–brain barrier (BBB) (Liebner et al., 2008; Jean LeBlanc et al., 2019). The BBB, which mainly exists in small arteries, capillaries, and veins, blocks circulating cells and molecules from the brain parenchyma and discharges metabolites or exotic substances to maintain the low permeability of the cerebrovascular system. Structurally, the BBB mainly refers to the layer of cerebrovascular endothelial cells in direct contact with blood, which in turn closely combines with perivascular cells, extracellular matrix membranes, astrocytes, and a small number of neurons. In the NVU, these cells and noncellular matrix components interact with endothelial cells, which play an important role in supporting and regulating BBB functions (Schaeffer and Iadecola, 2021).

The Wnt/β-catenin signaling pathway plays a critical regulatory role in regulating cerebrovascular development and BBB formation during embryonic development. This is determined by the following three aspects: 1) deficiency of the endothelial Wnt/β-catenin signaling pathway affects the development of cerebrovascular and BBB, but does not affect the development and function of other organs and tissues (Stenman et al., 2008; Daneman et al., 2009); 2) knockout of *Wnt7a/7b* (which shows the highest expression in the brain tissue) or receptor *Fzd4,2,7*, *LRP5/6*, receptor activator *GPR124* in endothelial cells, or *Ctnnb1* (β-catenin) can lead to abnormal cerebrovascular development and BBB function (Cullen et al., 2011; Wang et al., 2012; Zhou et al., 2014); and 3) upregulation of the Wnt signaling activity significantly upregulates the expression of BBB-function-related genes in cultured endothelial cells (Liebner et al., 2008).

In recent years, many studies have shown that the activity of the Wnt/β-catenin signaling pathway in ischemic brain tissue is significantly decreased in animal models of cerebral ischemia-reperfusion. Clinically, some genetic variants of *Lrp6* may be correlated with the risk of ischemic stroke (Calvier et al., 2022). Additionally, the levels of plasma DKK1 have been reported to be higher in patients with acute ischemic stroke than in healthy individuals (He et al., 2016; Zhu et al., 2019; Stavrinou et al., 2021). Systematic investigation of all types of cells in the brain affected by the downregulation of Wnt signaling is still lacking. However, studies have shown that in cerebrovascular endothelial cells, neurons, pericytes, astrocytes, microglia, and oligodendrocytes, Wnt signaling not only regulates their survival and proliferation but also affects their unique biological functions.

### Cerebrovascular Endothelial Cells

As the first barrier for peripheral tissue and blood components to enter the brain parenchyma, cerebrovascular endothelial cells are the core components of the BBB and have a series of special structural and molecular characteristics that determine the high selective permeability of the BBB. High expression of intercellular tight junction proteins is one of the main characteristics of cerebrovascular endothelial cells. Claudin-5, occludin, and scaffold protein ZO-1/2 are responsible for anchoring the former two proteins to the cytoskeleton (Zhao et al., 2015). A study has shown that the liposolator-induced lipoprotein receptor LSR (angulin-1) is specifically overexpressed in the BBB and acts as a tight junction protein between the three cells to enhance BBB function (Sohet et al., 2015).

Appropriately active Wnt signaling is essential for maintaining the BBB, both structurally and functionally. A variety of co-receptors on the surface of endothelial cells can affect signal transduction through interaction with Wnt receptors. For instance, Reck, a GPI-anchored membrane protein, and Gpr124, an orphan G-protein-coupled receptor, have been implicated in Wnt7a/Wnt7b mediated canonical Wnt signaling in the CNS vascular development and functional maintenance. Cho et al. showed that cerebral vascular endothelial cell-specific knockout of *Reck* impairs CNS angiogenesis and BBB integrity (Cho et al., 2017). Another study showed that the disruption of BBB integrity under acute brain ischemia/reperfusion (I/R) was significantly weakened in mice with conditional knockout of endothelial *Gpr124*, a Wnt7-specific coactivator of Wnt/β-catenin signaling, which could be rescued by genetic activation of endothelial β-catenin (Chang et al., 2017). A follow-up study showed that the variants of *Gpr124* and *Wnt7a* are associated with an increased risk of hemorrhagic transformation in patients with acute ischemic stroke after intravenous thrombolysis (Ta et al., 2021). Mechanistically, Reck binds with low micromolar affinity to the intrinsically disordered linker region of Wnt7. This process is manifested as the interaction between Gpr124 and Dishevelled, which aggregated Gpr124 with Reck-Wnt7 into Wnt/Frizzled/Lrp5/6 complex, resulting in increased local availability of Wnt7 for downstream signaling (Eubelen et al., 2018). Most recently, an engineered *Wnt7a* mutant lacking the C-terminal domain and an embedded Frizzled-contact site could retain partial but selective activity on the Gpr124/Reck-containing receptor complexes of endothelial cells. From a therapeutic standpoint, this artificial Wnt protein can specifically target Gpr124/Reck to repair the BBB in rodent ischemic stroke and glioblastoma models (Martin et al., 2022). The above studies define a modality to repair the BBB by reactivating the endothelial Wnt/β-catenin signaling, which, therefore, may have potential therapeutic value in other CNS diseases, such as multiple sclerosis, epilepsy, and Alzheimer’s disease.

The endothelial tight junctions and extracellular basal membrane ensure low passive transportation between blood and brain parenchyma. Apart from this, the profoundly low rate of transcytosis is also an important property of BBB. Although Wnt signaling has not been shown to influence transcytosis in BBB, in blood–retinal barrier (BRB), Wnt signaling directly regulate the transcription of an endothelium-specific transcytosis inhibitor called major facilitator superfamily domain-containing protein 2a (MFSD2A), in a Wnt/β-catenin-dependent manner. Mice lacking either the Lrp5 or the Wnt ligand Norrin exhibit increased retinal vascular leakage and enhanced endothelial transcytosis (Wang et al., 2020). Therefore, it can further be suggested that the Wnt/β-catenin signaling pathway possibly influences the CNS endothelium integrity by affecting the transcytosis mechanism as well (Wang et al., 2020; Yemanyi et al., 2021).

### Neuron

Wnt signaling pathway has been well-established to play a critical role in neural development, axonal outgrowth, synaptogenesis, fate decision, and survival (Lie et al., 2005; Kuwabara et al., 2009; Alves dos Santos and Smidt, 2011). Dysregulation of Wnt/β-catenin signaling has also been observed in many distinct pathologies, including hepatic fibrosis, tumor growth, and ischemic stroke (Mastroiacovo et al., 2009; Okamoto et al., 2011; Clevers and Nusse, 2012). However, whether Wnt/β-catenin signaling plays a role in the functional maintenance of mature neurons and changes under pathological conditions such as neuronal injury have not been thoroughly examined. It has been reported that sustained overexpression of Wnt by lentivirus ameliorates deficient motor behavior, and increases neuronal survival by promoting axon regeneration and inhibiting astrocytic scar formation in a spinal cord injury model (Suh et al., 2011). As for ischemic stroke, intranasal administration of Wnt-3a protein has been found to reduce cerebral infarction and neuronal apoptosis, which may be mediated through the dephosphorylation of GSK-3β, which in turn increases nuclear β-catenin and relieves overactive caspase-3 through Foxm1 after ischemia/reperfusion injury (Wei et al., 2017; Matei et al., 2018). Interestingly, the dephosphorylation of GSK-3β has been shown to influence the expression of apoptotic/cell death-related and survival/neurotrophic genes, which may contribute to the pro-neuronal survival effects of Wnt/β-catenin signaling (Tang et al., 2010).

Although Wnt3a protein-mediated Wnt/β-catenin signaling activation showed a decent neuronal effect, due to its hydrophobicity, Wnt3a can barely exert any biological function through systematic administration without a cosolvent, such as detergents (e.g., CHAPS) or solubilizers (e.g., MβCD), which makes it almost impossible to conduct clinical studies. Therefore, genetic engineering-based Wnt surrogates may be a promising strategy for the development of BBB protective drugs in the future.

### Oligodendrocytes

The white matter consists of axons, oligodendrocytes, and astrocytes, which are the most common injury sites for ischemic stroke (Qian et al., 2016). Neuronal axons are wrapped in myelin sheets, which are critical for the accuracy and speed of nerve signal conduction. Therefore, axonal damage is often accompanied by a reduction in myelin sheaths, known as demyelination, which accounts for the loss of oligodendrocytes.

To achieve remyelination after brain injury, oligodendrocytes must develop from oligodendrocyte precursor cells (OPCs). Wnt/β-catenin appears to play a crucial role in spatiotemporal regulation of oligodendrocyte differentiation (Garcia-Martin et al., 2021). A recent study employed transplantation of OPCs in a transient middle artery occlusion (MCAO) model and found significant functional angiogenesis and increased myelin basic protein expression (Wang et al., 2021c). Furthermore, this process is likely dependent on angiogenesis induced by Wnt7a-mediated activation of the Wnt/β-catenin signaling pathway.

### Microglia

As the dominant immune cells in the CNS, microglia are well-characterized for their secretory and phagocytic properties. More importantly, microglia express various immunological receptors, which endow them with a Janus face to function in both positive and negative manner towards neurons. For instance, triggering receptor expressed in myeloid cells 2 (TREM2) is a pattern recognition receptor expressed in myeloid cells, including microglia. TREM2 was found to prohibit β-catenin degradation, thereby activating the canonical Wnt pathway (Meilandt et al., 2020). Genetically, TREM2 mutations cause abnormalities in Wnt/β-catenin signaling and microglial overactivation, which in turn increases the risk of Alzheimer’s disease (Huang et al., 2022).

From the perspective of neurogenesis, microglia were found to selectively engulf synapses based on specific chemokine signals such as CR3/CX3CL1. CX3CL1 interacts with its receptor, fractalkine, specifically expressed on neurons, and thus activates microglia by phagocytosis (Cardona et al., 2006; Paolicelli et al., 2011). This process is important for maintaining an adequate number of synapses and to promote the formation of neuronal circuits. Interestingly, when the Wnt/β-catenin signaling pathway is suppressed in neurons, fractalkine expression decreases substantially, causing synapse degeneration. Therefore, Wnt signaling may play a role in microglial-involved synapse modification.

Furthermore, when the CNS is confronted with pathological conditions such as neurodegenerative diseases or ischemic stroke, the deleterious circumstance can enhance the combination of the complement fragment C1q and synapses, which causes the over-activation of microglial phagocytosis towards synapses and eventually damages the neuronal cells (Mercurio et al., 2022). Dying neurons undergo p53-mediated apoptotic signaling pathway, which leads to the expression of the downstream target gene *Dkk1* and further inactivates the Wnt/β-catenin signaling pathway (Wang et al., 2000). Meanwhile, deleterious substances from the eliminated synapses increase the delivery of inflammatory factors from microglia and further aggravate microglial inflammation and synapse damage.

### Astrocytes

**FIGURE 3:**
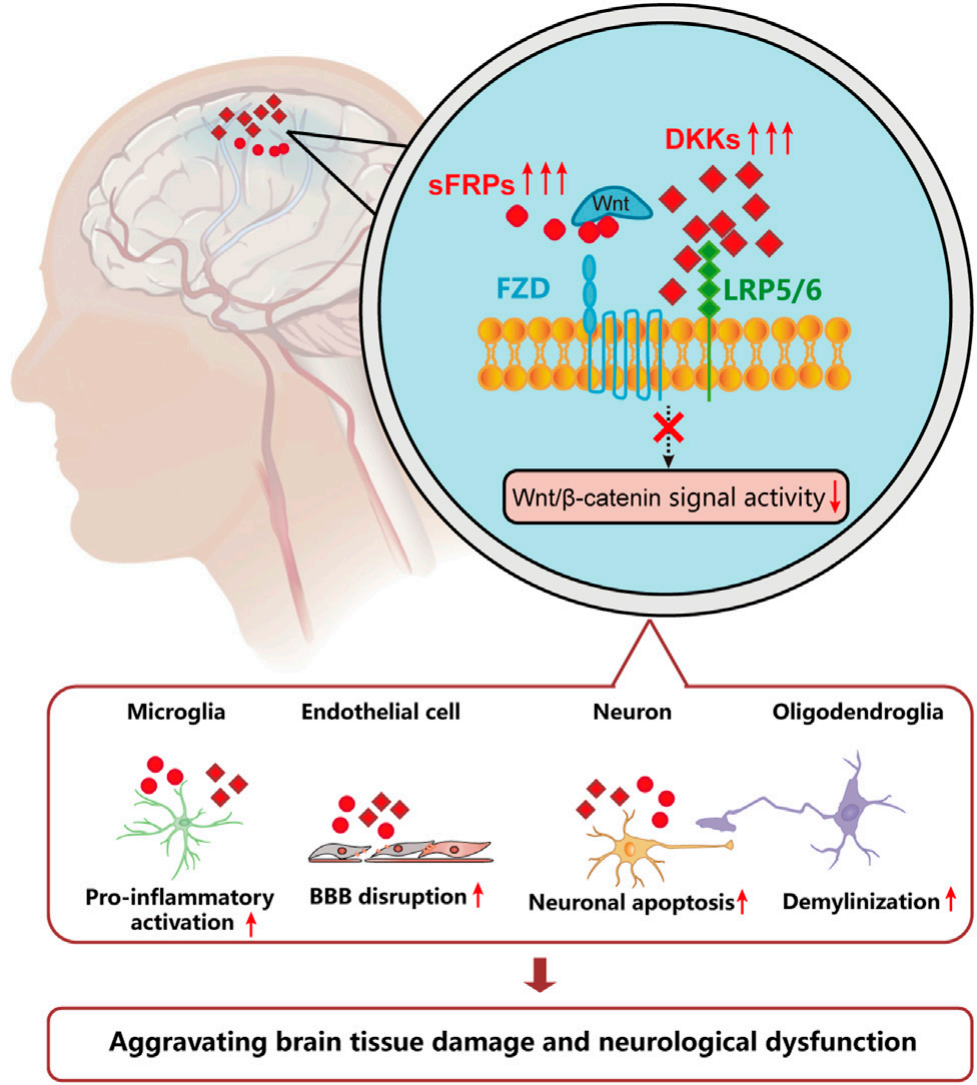
The potential implications of Wnt/β-catenin among cells from ischemic brain. After an ischemia incident, the microenvironment in ischemic brain leads to decreased activity of Wnt/β-catenin signaling pathway. The regulatory mechanisms of which include increased level of Dkks and sFRPs, which leads to increased BBB disruption, neuronal apoptosis, demyelination, and overactivation of microglia.

As a major component in CNS, astrocytes play an important role in maintaining brain function. Astrocytic abnormality has been observed in many CNS diseases, such as Alzheimer’s disease, multiple sclerosis, and hemorrhagic stroke. It has been shown that the receptor of Wnt7b, Frizzled-7 was widely expressed among cells in CNS, including endothelial cells, neurons, and astrocytes. In an experimental intracerebral hemorrhage model in mice, activation of Wnt signaling by Frizzled-7 modified by CRISPR substantially reduced cerebral edema, BBB leakage, and associated behavioral deficiency, while downregulated expression of Frizzled-7 markedly aggravated the above phenomenon. Further, it was found that the activation of Frizzled-7-mediated Wnt/β-catenin signaling mostly takes place in the perihematomal endothelial cells, neurons, and astrocytes (He et al., 2021). The potential implications of Wnt/β-catenin among cells from ischemic brain are depicted in Figure 3.

## Conclusion

The Wnt/β-catenin signaling pathway has been proved to be involved in a variety of physiological and pathological processes. More recently, several preclinical studies have found a decline in Wnt signaling activity after stroke onset, and activators of the Wnt/β-catenin signaling pathway have shown encouraging therapeutic effects. Current mechanisms of action aiming at stimulating the Wnt/β-catenin signaling pathway mainly include inhibitors of GSK-3β phosphorylation, engineered Wnt proteins, antagonists of Wnt inhibitors (DKKs, SFRs), and agonists towards the co-receptor of Wnt receptors (Table 1). However, extensive studies are needed to investigate the metabolic characteristics and safety of the protein molecules used. Lithium chloride is extensively used in clinical practice to treat bipolar mood disorders. Recently, lithium has also been used as an inhibitor of GSK-3β, which is a chemical activator of the Wnt/β-catenin signaling pathway. The administration of lithium exhibits a protective effect on BBB function, as observed in an experimental mice for ischemic stroke (Ji et al., 2021; Song et al., 2022). Therefore, future clinical studies are needed to evaluate the systematic effects and safety of targeting the Wnt/β-catenin signaling pathway for the treatment of ischemic stroke. Moreover, because BBB breakdown also occurs in metastatic encephaloma, leukemia, and toxic or metabolic encephalopathy, it is worthwhile to investigate the therapeutic potential of Wnt activators in diseases involving BBB dysfunction.

**TABLE 1:** Pharmacological agents target Wnt/β-catenin in experimental stroke.

Agents	Models	Proposed Mechanisms	References
lithium chloride	Transient MCAO in mice; Brain hemorrhage in mice	Inhibitor of GSK-3	Ji et al. (2021); Song et al. (2022)
TWS119	Permanent MCAO with hypoxia treatment in mice	Specific inhibitor of GSK-3β	Song et al. (2019)
6-bromoindirubin-3′-oxime	Transient MCAO with rtPA treatment in mice	Inhibitor of GSK-3	Jean LeBlanc et al. (2019)
Gpr124/Reck/Fz1	Transient MCAO in mice	Engineered Wnt7A fusion protein	Martin et al. (2022)
Wnt3a protein	Transient MCAO in mice	Wnt3a protein with cosolvent	Matei et al. (2018)
Wnt1 protein		Activation of Akt1	Chong et al. (2010)
Sulindac	Permanent MCAO in rat	upregulated the expression of Dvl, beta-catenin, and downregulated APC	Xing et al. (2012)
Dkk-1 antisense oligonucleotides	Permanent MCAO in mice		Cappuccio et al. (2005); Mastroiacovo et al. (2009)
